# NGS QC Toolkit: A Toolkit for Quality Control of Next Generation Sequencing Data

**DOI:** 10.1371/journal.pone.0030619

**Published:** 2012-02-01

**Authors:** Ravi K. Patel, Mukesh Jain

**Affiliations:** Functional Genomics and Bioinformatics Laboratory, National Institute of Plant Genome Research (NIPGR), New Delhi, India; Auburn University, United States of America

## Abstract

Next generation sequencing (NGS) technologies provide a high-throughput means to generate large amount of sequence data. However, quality control (QC) of sequence data generated from these technologies is extremely important for meaningful downstream analysis. Further, highly efficient and fast processing tools are required to handle the large volume of datasets. Here, we have developed an application, NGS QC Toolkit, for quality check and filtering of high-quality data. This toolkit is a standalone and open source application freely available at http://www.nipgr.res.in/ngsqctoolkit.html. All the tools in the application have been implemented in Perl programming language. The toolkit is comprised of user-friendly tools for QC of sequencing data generated using Roche 454 and Illumina platforms, and additional tools to aid QC (sequence format converter and trimming tools) and analysis (statistics tools). A variety of options have been provided to facilitate the QC at user-defined parameters. The toolkit is expected to be very useful for the QC of NGS data to facilitate better downstream analysis.

## Introduction

Next generation sequencing (NGS) technologies provide a revolutionary tool for numerous applications and are capable to generate several gigabases of sequence data in a single experimental run. These technologies are being increasingly used for various genome and transcriptome sequencing related applications due to their speed, cost-effectiveness and high-throughput nature [1], [2]. However, several sequence artifacts, including read errors (base calling errors and small insertions/deletions), poor quality reads and primer/adaptor contamination are quite common in the NGS data, which can impose significant impact on the downstream sequence processing/analysis. The quality of data is very important for various downstream analyses, such as sequence assembly, single nucleotide polymorphisms identification and gene expression studies. Most of the programs available for downstream analyses do not provide the utility for quality check and filtering of NGS data before processing. Therefore, these sequence artifacts need to be removed before downstream analyses, otherwise they may lead to erroneous conclusions.

The quality of data may be affected by several factors regardless of the NGS platform. Although the commercial vendors for all the sequencing platforms provide a quality control (QC) pipeline for filtering of sequencing output, several sequence artifacts still remain in the dataset. Therefore, it is advisable to perform QC and filtering of high-quality (HQ) sequencing data at the end-user level. For example, we rejected about 8% of the sequence reads obtained after filtering through QC pipelines of sequencing platforms, in our QC analysis of Illumina and Roche 454 data [3], [4]. A few online/standalone software packages/pipelines with different features have been developed for QC of NGS data [5]–[9]. Many of these are specific for a particular sequencing platform and have one or the other limitation(s). Therefore, there is still a need for the development of better tools with additional/better features.

In this study, we have developed a NGS QC Toolkit, comprised of various easy-to-use standalone tools for quality check and filtering, trimming, generating statistics and conversion between different file formats/variants of NGS data from Illumina and Roche 454 platforms. The toolkit allows automatic and fast parallel processing of large amount of sequence data with user-friendly options. Given the importance of QC of NGS data, we anticipate that this toolkit will be very useful for the sequencing based biological research.

## Results and Discussion

**Figure 1:**
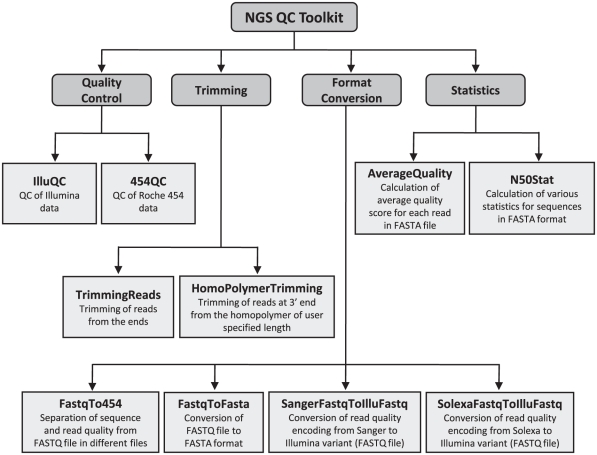
Flow chart showing various tools included in NGS QC Toolkit. The tools have been grouped into QC tools, trimming tools, format converters and statistics tools.

NGS QC Toolkit provides tools for QC of Illumina and Roche 454 data and additional tools for conversion between NGS data formats, sequence trimming and statistics calculation. Various tools available in the NGS QC Toolkit along with their utility have been summarized in Figure 1. All the tools are equipped with user-friendly options and provide proper guidelines for running. Various tools and their key features included in the toolkit are described below.

### QC tools for Illumina and Roche 454 sequencing data

**Figure 2:**
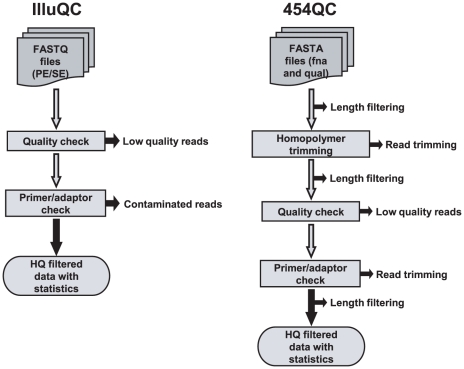
Workflow of the QC tools for Illumina (IlluQC) and Roche 454 (454QC) data. IlluQC tools process FASTQ files containing paired-end (PE) and/or single-end (SE) reads. After filtering low-quality reads and reads containing primer/adaptor contamination as per given criteria, high-quality (HQ) reads and QC statistics are generated in the output folder. 454QC tools process SE and PE sequence and quality files in FASTA format. After trimming reads containing homopolymer (optional), low-quality reads are removed and reads containing primer/adaptor contamination are trimmed as per given criteria. Each of these steps is followed by filtering of reads of given length cut-off. Finally, HQ reads and QC statistics are generated in the output folder.

**Figure 3:**
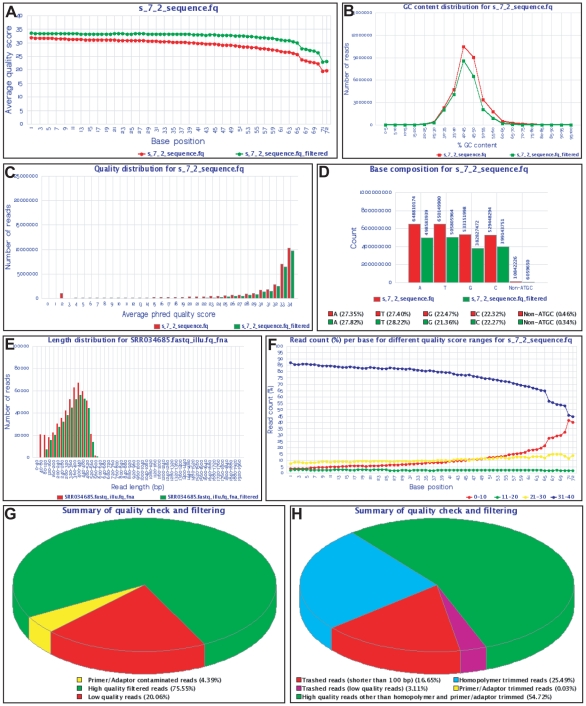
Snapshots showing graphs of various QC statistics generated as output by QC tools. (A) Average quality score for each base position, (B) GC content distribution, (C) Average Phred quality score distribution, (D) Base composition and (E) read length distribution for both input (red) and HQ filtered (green) data. (F) Percentage of reads with different quality score ranges at each base position. (G,H) Pie charts show summary of QC analysis of Illumina (G) and Roche 454 (H) data.

IlluQC and 454QC tools have been developed for QC of sequencing data generated from Roche 454 and Illumina platforms, respectively. These tools can accept sequencing data in various formats as input and perform quality check using default/user-defined parameters. At the end, QC reports for unfiltered (input) and filtered (output) data are generated in different formats along with filtered HQ data files as output. A schematic representation of the workflow for QC tools has been depicted in Figure 2. Briefly, IlluQC (IlluQC.pl and IlluQC_PRLL.pl) tools can auto detect the FASTQ variant of the input file(s) and process both paired-end (PE) and single-end (SE) sequencing data for QC. These tools set the quality scoring system according to the FASTQ variant [10] and perform quality check using parameters provided by the user. The reads having at least given number of bases (% read length) with more than or equal to the specified Phred quality score are filtered as HQ reads. First and last twenty bases of primer/adaptor sequences (user-defined) are matched with the HQ reads allowing single bp mismatch. The reads containing primer/adaptor sequences are discarded. Likewise, 454QC (454QC.pl, 454QC_PE.pl and 454QC_PRLL.pl) tools perform QC of Roche 454 sequencing data in FASTA format (fna and qual files). These tools filter reads shorter than given length cut-off at several steps, trim reads containing homopolymer (optional) and filter HQ reads based on Phred quality score (provided in qual file). The use of optional parameter, homopolymer trimming, trims the reads containing homopolymers from the first base of homopolymer (of user-specified length) up to the end of sequence. First and last 20 bases of primer/adaptor sequences (user-specified) are matched with first and last 50 bases of the HQ reads. The reads showing match allowing only single bp mismatch are trimmed at their respective end(s). Finally, HQ filtered data is exported with the detailed QC statistics for each step of processing for both input and filtered data in the form of text files and graphs (Fig. 3, Figure S1) along with a consolidated HTML report file in both the QC tools. The tool for QC of PE Roche 454 data, 454QC_PE.pl, identifies linker sequence to separate the PE reads as the first step. The following steps of QC on these PE reads and unpaired reads (where linker sequence could not be identified) are same as other 454QC tools (Fig. 3, Figure S1).

### Key features of QC tools

#### Parallelization

Time required to process huge amount of NGS data needs to be minimized using optimal computational resources. To achieve this goal, we have implemented parallelization in QC tools in two ways; multiprocessing (IlluQC.pl and 454QC.pl) and multithreaded (IlluQC_PRLL.pl and 454QC_PRLL.pl). Multiprocessing has been implemented to process multiple files in parallel using QC tools, where one CPU is allocated to each file (on multi-CPU system). In multithreaded tools, single file is divided into parts and processed in parallel using multiple CPUs (on multi-CPU system) and merged at the end. However, these tools can also run on single CPU system efficiently without any additional requirements. For machines having a single CPU, the use of multiprocessing tools is recommended for QC; otherwise the choice of multithreaded tools will be better for processing huge amount of NGS data.

#### Paired-end data processing

While filtering of HQ reads from PE data, it is very crucial to maintain the pairing information, which is important for several downstream analyses. IlluQC processes both (forward and reverse) reads simultaneously and exports HQ filtered reads in a separate file, only if both of them pass the filter criteria, thus keeping the pairing information intact. In addition, it also exports one of paired reads as HQ filtered output in a separate file, if it passes the filter criteria. This un-paired HQ filtered data can be used along with PE data as SE reads for various studies. In this way, the QC tools try to retain all important HQ sequencing data after removal of sequence artifacts. To our knowledge, other tools do not provide an option to process PE files together, which leads to the loss of pairing information, important for specific analyses, such as *de novo* assembly and read mapping.

#### Homopolymer trimming

In Roche 454 sequencing, the encounter of homopolymers is a major problem. The signal intensity distribution broadens with the length of the homopolymer, resulting in an ambiguous base call [11], which may lead to frame-shift affecting the downstream processing. An optional parameter has been provided in 454QC tools to trim the reads containing homopolymer(s) of user-specified length from 3′-end, which will take care of the limitation associated with Roche 454 sequencing. However, it is a highly stringent parameter and should be used carefully as per requirement of downstream analysis to avoid the loss of important sequence data.

#### Auto detection of FASTQ variant

Due to lack of awareness about different variants of FASTQ format (Sanger, Solexa and Illumina) [10], it is difficult for users to identify them and use appropriate tool for quality check as the quality score encoding varies among different variants. We have implemented a feature in IlluQC to detect the variant of input FASTQ file automatically and set the quality scoring system accordingly for QC. However, sometimes problem may arise in the auto detection of FASTQ variant due to the quality values falling into ambiguous score range. To overcome such problem, user can also specify the FASTQ variant, if known.

#### Primer/adaptor contamination removal

Most publicly available programs for QC of NGS data do not provide the option for primer/adaptor contamination removal. A list of standard sequencing assays (six different assays for Illumina and four for Roche 454 sequencing) and primer/adaptor sequences used in them are provided in the QC tools. Tools utilize the standard primer/adaptor sequences for selected assay and remove (IlluQC) or trim (454QC) the contaminated reads. Furthermore, considering the utility of toolkit for QC of data generated using non-standard sequencing protocols, an option has been provided to filter the non-standard primer/adaptor sequences (user-provided) as well.

#### Processing compressed files

One of the major problems associated with NGS is data storage and handling. Generally the huge amount of data produced by NGS technologies is handled in the form of compressed files (gzip) to lower the requirement of storage space and time for data transfer. Several downstream analysis tools can also use compressed files as input, and to decompress the data just for the QC step is inutile. Our QC tools support reading and writing of compressed NGS data files (gzip). An option has been provided to specify whether to get the output HQ filtered data in compressed form.

### Input and output of QC tools

Input to these tools is sequence data in FASTQ (IlluQC) or FASTA (454QC) format with various command line options to provide control over QC. All parameters except for input files are optional and set to the sensible default values, which can be viewed by running tools without any arguments (or using -h option). Quality statistics of the input and output filtered data is generated in the form of text files (Figure S1) and graphs (Fig. 3), and HQ filtered data is exported in the output folder. In addition, a consolidated QC report is generated in the HTML format. The statistics in text files can be exported as formatted text or tab-delimited columns, which include parameters used, number and percentage of reads filtered at each step of QC and statistics for input and filtered data including minimum, maximum, average, median and N50 read length, number and percentage of reads, total bases, total HQ bases and non-ATCG bases (Figure S1). The graphs generated by these tools represent various QC statistics, including the average quality scores at each base position for both input and filtered reads to compare and check the overall quality improvement after filtering (Fig. 3A), percentage GC content distribution (Fig. 3B), average quality distribution (Fig. 3C), total base count (Fig. 3D) and read length distribution (Fig. 3E) for both input and filtered reads, and percentage of filtered reads with different quality score ranges at each base position (Fig. 3F). A pie chart is also generated for the summary of quality check and filtering analysis showing number and percentage of filtered reads at each step (Fig. 3G,H). In addition to the output files, tools also print messages on the command console for the variant of input FASTQ file (for IlluQC), steps and progress of the analysis with number and percentage of reads processed. Altogether, QC tools generate various statistics and publication ready graphs of QC analysis along with HQ filtered data as output.

### Performance

The QC tools provide efficient and dedicated tools for the quality check and filtering of NGS data. As described above, two versions of QC tools for Illumina (IlluQC.pl and IlluQC_PRLL.pl) and Roche 454 data (454QC.pl and 454QC_PRLL.pl) have been developed. We have validated the performance of the QC tools using more than 20 datasets downloaded from NCBI short read archive (SRA) public database and our own datasets. A few examples of case studies showing significant improvement in the quality of filtered data using NGS QC toolkit have been provided in Data File S1. Both versions of QC tools perform identical analysis but differ in time and resource requirements (IlluQC_PRLL.pl and 454QC_PRLL.pl require less time utilizing more resources). For example, Illumina dataset (SRR094181, more than 12.6 million 50 bp PE reads accounting for ∼5 GB data) could be processed in 115 min utilizing ∼12 MB of memory by IlluQC.pl, whereas IlluQC_PRLL.pl took only 32 min on six CPUs using ∼1.5 GB of memory. Roche 454 dataset (SRR034685, more than 0.5 million reads) could be processed in 11 min using a maximum of 71 MB memory by 454QC.pl. However, 454QC_PRLL.pl took only 3 min to process the same data on six CPUs using maximum of 1.0 GB memory. The specified time and memory requirements may vary considerably for machines with different type of processors and architecture. The memory requirement is not dependent on the amount of input data for multiprocessing tools. These tools utilize minimum amount of memory required to store the information necessary to calculate statistics and store a single read at a time. Multithreaded tools could process data at much faster speed than multiprocessing tools. Furthermore, the speed of QC analysis can be elevated by providing more computational resources using high-end workstation/server with multi-core processors and high random access memory.

### Additional tools

#### Sequence format converters

SangerFastqToIlluFastq.pl and SolexaFastqToIlluFastq.pl convert the fastq-sanger and fastq-solexa variants, respectively, to fastq-illumina variant of FASTQ format using the equations described previously [10]. FastqTo454.pl and FastqToFasta.pl separate sequences and their quality from the input FASTQ file (auto-detection of the variant) and print them in separate FASTA files.

#### Sequence trimming tools

Generally, it has been observed that quality of few bases at the end(s) of reads is substantially lower as compared to other bases. Sometimes, even the QC tools are not able to filter the reads containing such low-quality bases due to their overall HQ. In addition, it is not advisable to discard the whole read due to lower quality of only few bases at the ends. Therefore, it is crucial to trim such bases before any downstream analysis. TrimmingReads.pl performs trimming in two ways. First, it trims fixed (user-specified) number of bases from 5′ and/or 3′ end of the reads and corresponding qualities from the input FASTQ file. Second, it trims low quality bases from 3′ end of the read using user-defined threshold value of quality score. Another tool, HomopolymerTrimming.pl, searches for homopolymer of user-specified length and trims the 3′ end of the read from the first base of homopolymer. This option has been provided as a part of the 454QC tool also and may be used independently.

#### Statistics tools

These tools analyze FASTA format file to calculate different sequence statistics. AvgQuality.pl requires qual file (quality in FASTA format) and calculates the average quality score for each read and overall average quality score for all the reads. N50Stat.pl takes FASTA sequence file to calculate various statistics, such as total number of reads/sequences in the file, total and individual (A,T,C,G and N) number of bases, G+C and A+T counts, and minimum, maximum, average, median, N25, N50, N75, N90 and N95 length. These tools will facilitate non-experts to assess various sequence statistics.

### Quick comparison with existing tools

Only a few standalone tools for QC of NGS data are publicly available other than commercial softwares supplied with the sequencing machines, which are not sufficiently optimal. Different tools with diverse features have been developed based on different concepts and algorithms. Many of these tools are sequencing platform-specific and/or are meant for a specific step of QC (Table 1). For example, TagDust program was developed to eliminate the reads generated from Illumina platform showing matches to library sequences [12]. The pipeline, PIQA, was proposed as an extension of the standard Illumina pipeline for the identification of various technical problems, such as defective files, mistakes in sample/library preparation and abnormalities in frequencies of sequenced reads [5]. ShortRead also provides the utility of quality assessment and filtering of Illumina data [13]. The online data analysis platform, Galaxy, provides several tools to manipulate and analyze the FASTQ data [6]. CANGS is capable of filtering low quality sequences, singletons, removing primers and identifying barcodes from Roche 454 data [14]. TagCleaner v0.11 identifies and removes various tags from the Roche 454 data [8]. However, our NGS QC Toolkit provides dedicated tools for QC of Illumina and Roche 454 data with additional tools for handling/processing NGS data. A comparison of the features provided by different tools, including TagDust [12], CANGS [14], TagCleaner [8], PRINSEQ [9], SolexaQA [7], FastQC, FASTX-Toolkit and NGS QC Toolkit (this study) is given in Table 1. Although a few features of other tools are not available in the current version of NGS QC toolkit, several additional and better features have been provided for QC analysis. For example, to our knowledge, none of the other tools ensure the integrity of PE data. Only FastQC and our toolkit support parallelization to speed up the processing of large sequence data. Like our toolkit, SolexaQA also requires GD modules for statistics in graphical form, however, the requirement of R installation is additional, which may be difficult to non-experts. Furthermore, the use of web interface is time consuming due to uploading and downloading huge NGS data [6], [8], [9]. FastQC reports only the quality and statistics of sequence data without HQ filtering. However, these tools include some additional functionality and perform the task they are meant for and may improve on their limitations in future.

**Table 1:** Comparison of various features of NGS QC toolkit and other available QC tools.

Feature\Tools	NGS QC Toolkit v2.2	FastQC v0.10.0	PRINSEQ-lite v0.17^1^	TagDust	FASTX-Toolkit v0.0.13	SolexaQA v1.10	TagCleaner v0.12^1^	CANGS v1.1
Supported NGS platforms	Illumina, 454	FASTQ^2^	Illumina, 454	Illumina, 454	Illumina	Illumina	Illumina, 454	454
Parallelization	Yes	Yes	No	No	No	No	No	No
Detection of FASTQ variants	Yes	Yes	Yes	No	No	Yes	No	No
Primer/Adaptor removal	Yes	No^3^	No	Yes	Yes	No	Yes^4^	Yes
Homopolymer trimming (Roche 454 data)	Yes	No	No	No	No	No	No	Yes
Paired-end data integrity	Yes	No	No	No	No	No	No	No
QC of 454 paired-end reads	Yes	No	No	No	No	No	No	No
Sequence duplication filtering	No	No^5^	Yes	No	Yes	No	No	Yes
Low complexity filtering	No	No	Yes	No	Yes	No	No	No
N/X content filtering	No	No^6^	Yes	No	Yes	No	No	Yes
Compatability with compressed input data file	Yes	Yes	No	No	No	No	No	No
GC content calculation	Yes	Yes	Yes	No	No	No	No	No
File format conversion	Yes	No	No	No	No	No	No	No
Export HQ and/or filtered reads	Yes	No	Yes	Yes	Yes	No	Yes	Yes
Graphical output of QC statistics	Yes	Yes	No^7^	No	Yes	Yes	No^7^	No
Dependencies	Perl modules: Parallel::ForkManager, String::Approx, GD::Graph (optional)	-	-	-	Perl module: GD::Graph	R, matrix2png	-	BLAST, NCBI nr database

1 Standalone version.

2 Data of any platform in FASTQ file format.

3 only detection.

4 only one primer/adaptor sequence at a time.

5 only reports duplication and that too is for only first 200,000 reads.

6 only reports N/X content.

7 yes, in case of online version.

The scale and efficiency of sequencing can provide unprecedented progress in the genomics, transcriptomics and epigenomics studies. The power of NGS technologies is increasingly being harnessed in various applications to address diverse range of biological problems. However, the quality of NGS data is very crucial in downstream analysis and its biological interpretation. We have developed an open source user-friendly toolkit for QC of Illumina and Roche 454 sequencing data, which has several additional features as compared to existing tools. QC tools are capable of processing multiple files in parallel very fast with optimal computational requirements and generating publication ready graphs for various QC statistics. Tools can process and generate compressed data files which significantly reduces disk storage requirement and are compatible with several downstream analysis tools. We anticipate that the toolkit will be very helpful for optimal QC of NGS data for downstream analysis.

## Methods

### Implementation

All tools in the toolkit have been developed using Perl programming language by implementing modularized structure using several sub-routines for various tasks, which allows better maintainability. GD module has been used to generate various graphs for statistics and String::Approx module for searching primer/adaptor sequence in input reads. Parallel::ForkManager and Threads modules have been utilized for parallelizing the QC tools. IO::Zlib module has been used to facilitate reading/writing compressed (gzip) files. QC reports are generated using Hypertext Markup Language (HTML) and Cascading Style Sheets (CSS). All the tools have been tested on Windows and Linux (CentOS) operating systems for full functionality.

### Availability, installation and usage

NGS QC Toolkit is a standalone and open source application freely available at http://www.nipgr.res.in/ngsqctoolkit.html. Detailed description about installation and usage of the toolkit is available in user manual on the web site. In brief, to install the toolkit, user needs to download NGSQCToolkit_v2.2.zip (current version) file from the website and unzip it. Dependencies for using toolkit include Perl interpreter (usually supplied with OS for Linux and ActivePerl for Windows) and additional Perl modules, GD (optional; required to generate QC graphs) and String::Approx. Their installation instructions can be found on their respective websites.

Different tools available in the toolkit, categorized on the basis of task they are meant to perform (Fig. 1), are available in different folders. These tools could be run using “perl <tool name> <options>” command on the command-line prompt. The parameter options for the input sequence data, QC analysis, processing and output, and their default settings can be viewed by using “-h” option in the above mentioned command. To check whether dependencies are resolved, QC tools can be run without any parameter, which will result into appropriate error/warning messages for the missing modules, if any. To input PE data into IlluQC, user needs to use “-pe” (“-se” for single-end data) option, followed by forward and reverse end read files, sequencing assay and FASTQ variant. In 454QC, “-i” option is used followed by read and quality files in FASTA format and sequencing assay. A switch “-p” is provided in IlluQC.pl and 454QC.pl to specify number of files to be processed simultaneously in parallel. Number of CPUs can be specified in multithreaded tools using switch “-c”. By default, tools generate output where the input files are located. Test data has been provided for download on the website, which include input data for Illumina (PE and SE) and Roche 454 platforms and output data from QC, trimming and statistics tools.

### Major enhancements

First version (v1.0) of the NGS QC toolkit included QC tools (IlluQC.pl and 454QC.pl) with basic functionality of quality check and primer/adaptor contamination removal for Illumina and Roche 454 data generating textual QC statistics, and sequence statistics analysis tools. In a major update of the toolkit (v2.0), parallelization was introduced in the QC tools to speed up the analysis. In addition, the feature of generating QC statistics in the form of graphs was implemented. We have also added the feature of reading/writing of compressed files (gzip) and generating consolidated QC report in HTML format in our earlier update (v2.1). Recently, IlluQC tools were updated to generate a graph depicting percentage of reads falling into different quality score ranges at each base position, TrimmingReads tool was modified to provide an additional option for trimming reads based on quality score and a new tool has been incorporated for the QC of Roche 454 paired-end data in the current version (v2.2).

## Supporting Information

Figure S1 Tables showing statistics generated by IlluQC (A) 454QC (B) and 454QC_PE (C) tools for QC analysis of Illumina, Roche 454 and Roche 454 PE data, respectively. Three tables are generated by the QC tools. First, Parameters; input parameters used for QC. Second, QC statistics; number and percentage of reads filtered at each step of QC analysis. Third, Detailed QC statistics; detailed statistics of both input and HQ filtered data for comparison.
(PDF)

Data File S1 Representative examples of case studies for QC of Illumina and Roche 454 data using NGS QC toolkit.
(PDF)
