# Supplementary material for: NGS QC Toolkit: A Toolkit for Quality Control of Next Generation Sequencing Data
Source: PLoS One. 2012 Feb 1;7(2):e30619. doi: 10.1371/journal.pone.0030619 (PMC3270013; doi:10.1371/journal.pone.0030619)
Supplement: Figure S1 — Tables showing statistics generated by IlluQC (A) 454QC (B) and 454QC_PE (C) tools for QC analysis of Illumina, Roche 454 and Roche 454 PE data, respectively. Three tables are generated by the QC tools. First, Parameters; input parameters used for QC. Second, QC statistics; number and percentage of reads filtered at each step of QC analysis. Third, Detailed QC statistics; detailed statistics of both input and HQ filtered data for comparison. (PDF) [file pone.0030619.s001.pdf]

Figure S1 Tables showing statistics generated by IlluQC (A), 454QC (B) and 454QC_PE (C) tools for QC analysis of Illumina, Roche 454 SE and Roche 454 PE data, respectively. Three tables are generated by the QC tools. First, Parameters; input parameters used for QC. Second, QC statistics; number and percentage of reads filtered at each step of QC analysis. Third, Detailed QC statistics; detailed statistics of both input and HQ filtered data for comparison.

**Parameters**	
Library type	Paired-end
Input files	s_7_1_sequence.fq<br>s_7_2_sequence.fq
Primer/Adaptor library	Paired End DNA Library
Cut-off read length for HQ	70%
Cut-off quality score	20
Only statistics	Off
Number of CPUs	20

**QC statistics**		
File name	s_7_1_sequence.fq	s_7_2_sequence.fq
Total number of reads	32950036	32950036
Total number of HQ reads	26340664	26340664
Percentage of HQ reads	79.94%	79.94%
Total number of bases	2372402592	2372402592
Total number of bases in HQ reads	1896527808	1896527808
Total number of HQ bases in HQ reads	1843456333	1824729397
Percentage of HQ bases in HQ reads	97.20%	96.21%
Number of Primer/Adaptor contaminated HQ reads	1445931	0
Total number of HQ filtered reads	24894733	24894733
Percentage of HQ filtered reads	75.55%	75.55%

**Detailed QC statistics**				
File name	s_7_1_sequence.fq	s_7_2_sequence.fq	s_7_1_sequence.fq_filtered	s_7_2_sequence.fq_filtered
Minimum read length	72	72	72	72
Maximum read length	72	72	72	72
Average read length	72	72	72	72
Total number of reads	32950036	32950036	24894733	24894733
Total number of reads with non-ATGC bases	156266	7121131	36143	5372421
Percentage of reads with non-ATGC bases	0.47%	21.61%	0.15%	21.58%
Total number of bases	2372402592	2372402592	1792420776	1792420776
Total number of HQ bases	2117658677	2064808038	1743636443	1727137632
Percentage of HQ bases	89.26%	87.03%	97.28%	96.36%
Total number of non-ATGC bases	2149470	10842226	77827	6059650
Percentage of non-ATGC bases	0.09%	0.46%	0.00%	0.34%

(A) IlluQC statistics

Parameters	
Input files	SRR034685.fastq_illu.fq_fna SRR034685.fastq_illu.fq_qual
Primer/Adaptor library	Rapid Library (Standard)
Homopolymer trimming	On
Length of the homopolymer to be removed	8
Length filter	On
Cut-off for minimum read length	100
Cut-off read length for HQ	70%
Cut-off quality score	20
Only statistics	Off
Number of CPUs	20

QC statistics	
File name	SRR034685.fastq_illu.fq_fna
Total number of reads	505332
Total number of trimmed reads containing homopolymer	128795
Total number of trashed reads (<100 bp in length after trimming)	84133
Total number of low quality reads (excluding <100 reads)	15734
Total number of HQ reads	405467
Percentage of HQ reads	80.24%
Total number of bases	173871662
Total number of bases in HQ reads	145562544
Total number of HQ bases in HQ reads	125010251
Percentage of HQ bases in HQ reads	85.88%
Number of Primer/Adaptor trimmed reads	168
Total number of HQ filtered reads	405465
Percentage of HQ filtered reads	80.24%

Detailed QC statistics		
File name	SRR034685.fastq_illu.fq_fna	SRR034685.fastq_illu.fq_fna_filtered
Total number of reads	505332	405465
Minimum read length	40	100
Maximum read length	1957	608
Average read length	344.07	358.99
Median read length	368	375
N25 length	472	470
N50 length	410	409
N75 length	333	332
N90 length	244	250
N95 length	191	205
Total number of bases	173871662	145558752
Total number of HQ bases	143958637	125007157
Percentage of HQ bases	82.80%	85.88%
Average quality score (Overall)	30.12	31.43

(B) 454QC statistics

**Parameters**	
Input files	SRR001355.fna<br>SRR001355.qual
Primer/Adaptor library	Rapid Library (Standard)
Homopolymer trimming	On
Length of the homopolymer to be removed	8
Length filter	On
Cut-off for minimum read length	50
Cut-off read length for HQ	70%
Cut-off quality score	20
Only statistics	Off
Number of processes	1

Detailed QC statistics

File name	SRR001355.fna	SRR001355.fna_filtered
Total number of reads	256503	255350
Minimum read length	40	25
Maximum read length	348	341
Average read length	248.22	238.09
Median read length	262	255
N25 length	277	274
N50 length	265	260
N75 length	251	240
N90 length	231	198
N95 length	188	172
Total number of bases	63670010	60796675
Total number of HQ bases	58364378	55872725
Percentage of HQ bases	91.67%	91.90%
Average quality score (Overall)	27.04	27.05

QC statistics

File name	SRR001355.fna	
Total number of reads	256503	
QC analysis of Paired reads:	Paired	(Read1 / Read2)
Total number of Paired reads	81708	(81708 / 81708)
Total number of trimmed reads containing homopolymer	3	(362 / 535)
Total number of trashed reads (<50 bp in length after trimming)	550	(20819 / 15759)
Total number of low quality reads (excluding <50 reads)	0	(0 / 190)
Total number of HQ reads	45490	(60889 / 65759)
Percentage of HQ reads	55.67%	(74.52% / 80.48%)
Total number of bases	18416651	(8698504 / 9718147)
Total number of bases in HQ reads	10413850	(8144697 / 9253019)
Total number of HQ bases in HQ reads	9659183	(7654312 / 8491176)
Percentage of HQ bases in HQ reads	92.75%	(93.98% / 91.77%)
Number of Primer/Adaptor trimmed reads	0	(13 / 4)
Total number of HQ filtered reads	45490	(60889 / 65759)
Percentage of HQ filtered reads	55.67%	(74.52% / 80.48%)
Total number of HQ filtered reads (Unpaired)	35668	
Percentage of HQ filtered reads (Unpaired)	43.65%	

QC analysis of Unpaired (UPOri) reads:	Total
Total number of Unpaired reads	174795
Total number of trimmed reads containing homopolymer	1762
Total number of trashed reads (<50 bp in length after trimming)	591
Total number of low quality reads (excluding <50 reads)	12
Total number of HQ reads	174192
Percentage of HQ reads	99.66%
Total number of bases	41634226
Total number of bases in HQ reads	41387871
Total number of HQ bases in HQ reads	38034881
Percentage of HQ bases in HQ reads	91.90%
Number of Primer/Adaptor trimmed reads	26
Total number of HQ filtered reads (Unpaired)	174192
Percentage of HQ filtered reads (Unpaired)	99.66%

Number of HQ filtered reads (Paired)	45490	(17.73%)
Number of HQ filtered reads (UPPair*)	35668	(13.91%)
Number of HQ filtered reads (UPOri*)	174192	(67.91%)

Total number of HQ filtered reads	255350	(99.55%)

* UPOri: Unpaired reads (i.e. reads without linker sequence) found in the input file
UPPair: One of the paired reads which passed QC

(C) 454QC_PE statistics
