# Supplementary material for: NGS QC Toolkit: A Toolkit for Quality Control of Next Generation Sequencing Data
Source: PLoS One. 2012 Feb 1;7(2):e30619. doi: 10.1371/journal.pone.0030619 (PMC3270013; doi:10.1371/journal.pone.0030619)
Supplement: Data File S1 — Representative examples of case studies for QC of Illumina and Roche 454 data using NGS QC toolkit. (PDF) [file pone.0030619.s002.pdf]

Data File S1. Representative examples of case studies for QC of Illumina and Roche 454 data using NGSQC toolkit.

Several datasets in different FASTQ variants and FASTA files were downloaded from NCBI SRA database, and analyzed using QC tools. The statistics showed significant improvement in the quality of filtered data as compared to the input data in most of cases. We have successfully utilized the toolkit for QC of Illumina and Roche 454 data in our recent studies for *de novo* assembly and characterization of chickpea transcriptome [1,2]. A few examples of case studies are given below.

1. Roche 454 data downloaded from the SRA accession number, SRR034685, was converted to the FASTQ format using “fastq-dump” tool of the “sratoolkit” provided by SRA, which generated sanger variant of FASTQ format. Roche 454 data in FASTQ file was converted to Roche 454 format using our FastqTo454.pl tool. QC analysis of this data using 454QC generated HQ filtered data and quality statistics. 26% of reads containing homopolymers of length greater than 7 bp were trimmed. Reads of low-quality and shorter than 100 bp accounting for 3% and 17% of total reads, respectively, were trashed and finally 80% of HQ reads were filtered.

2. Illumina data from SRA accession number, SRR094181, was converted to fastq-sanger variant of FASTQ format using tools of SRA toolkit. As IlluQC can detect any FASTQ variant, these FASTQ files were subjected to QC analysis directly and HQ filtered data with various statistics was exported. At default parameters, IlluQC trashed 8.9% low quality and 2% reads containing primer/adaptor sequences and exported rest of 89% reads as HQ filtered data.

3. Recently, we used both PE (SRR063783) and SE (SRR063784 and SRR063785) read Illumina data, totaling about 135 million reads, for *de novo* transcriptome assembly, which was pre-processed using NGS QC toolkit [1]. More than 8% of the reads did not meet our quality filter criteria and were discarded. Further, the quality statistics graph showed decrease in quality values for few bases at the 3’ end, which were trimmed using TrimmingReads.pl and used for *de novo* assembly. The validation of assemblies showed better assembly results with filtered data obtained after QC and trimming of reads as compared to filtered untrimmed data [1].

1. Garg R, Patel RK, Tyagi AK and Jain M (2011) *De novo* assembly of chickpea transcriptome using short reads for gene discovery and marker identification. DNA Res 18: 53-63.
2. Garg R, Patel RK, Jhanwar S, Priya P, Bhattacharjee A, et al. (2011) Gene discovery and tissue-specific transcriptome analysis in chickpea with massively parallel pyrosequencing and web resource development. Plant Physiol 156: 1661-1678.
